# Effectiveness of Educational Poster on Knowledge of Emergency Management of Dental Trauma - Part 2: Cluster Randomised Controlled Trial for Secondary School Students

**DOI:** 10.1371/journal.pone.0101972

**Published:** 2014-08-05

**Authors:** Cecilia Young, Kin Yau Wong, Lim K. Cheung

**Affiliations:** 1 Private Practice, Hong Kong; 2 Department of Biostatistics, University of North Carolina at Chapel Hill, Chapel Hill, North Carolina, United States of America; 3 The University of Hong Kong, Hong Kong; University of Toronto, Canada

## Abstract

**Objective:**

To investigate the effectiveness of educational poster on improving secondary school students' knowledge of emergency management of dental trauma.

**Methods:**

A cluster randomised controlled trial was conducted. 16 schools with total 671 secondary students who can read Chinese or English were randomised into intervention (poster, 8 schools, 364 students) and control groups (8 schools, 305 students) at the school level. Baseline knowledge of dental trauma was obtained by a questionnaire. Poster containing information of dental trauma management was displayed in a classroom for 2 weeks in each school in the intervention group whereas in the control group there was no display of such posters. Students of both groups completed the same questionnarie after 2 weeks.

**Results:**

Two-week display of posters improved the knowledge score by 1.25 (p-value = 0.0407) on average.

**Conclusion:**

Educational poster on dental trauma management significantly improved the level of knowledge of secondary school students in Hong Kong.

**Trial Registration:**

HKClinicalTrial.com HKCTR-1343

ClinicalTrials.gov NCT01809457

## Introduction

Prevalence of traumatic dental injuries of primary and permanent teeth is high throughout the world. Statistics from most countries showed that one fourth of all school children and almost one third of adults had suffered trauma to the permanent dentition, with variations among and within countries [1].

Early management is crucial to the prognosis for some dental injuries, especially avulsion [2]. However, most studies showed that teachers or school staff [3]–[15], parents [15]–[21], nurses [15], [22], [23], paramedics [23] and coaches [21] lacked the knowledge to manage traumatic dental injuries appropriately before the injured person reached dental professionals.

Since immediate management of traumatic dental injury does not require special skill but only knowledge, it can be performed by a lay person if one knows the procedures. The ideal situation is that such knowledge becomes everyone's basic practical knowledge. The earlier one learns the appropriate procedure, the higher chance one can save more traumatized teeth. In a literature search conducted before the study and finalized on Sept 21, 2013, there were only 4 studies investigating children's and teenagers' knowledge of traumatic dental injuries [24]–[27]. The results were that the subjects did not possess adequate knowledge.

Literature search of published studies on education in traumatic dental injuries prior to Sept 21, 2013 using keywords (“promotion*” or “intervention” or “education” or “knowledge” or “campaign” or “seminar” or “lecture” or “pamphlet” or “leaflet” or “banner” or “poster”) and (“dental injur*” or “traumatic dental injur*” or “dental trauma”) on Pubmed, Ovid, Web of Science and Cochrane Central Register of Controlled Trials resulted in only 14 papers related to education [28]–[41]. All of them targeted on adults, and there was no information about education in dental trauma for children or teenagers.

All Hong Kong primary school students (US Grade 1–6) are eligible to join the School Dental Care Service and most of them joined voluntarily. Every participant received a handbook for recording dental visits and it contained around 20 pages of dental health information. Since 1994, the handbook contained one page about avulsion and it mentioned that avulsed tooth should be put back into the socket, stored in milk or in mouth. However, in 2011, a survey about the level of knowledge of dental trauma revealed that such knowledge of secondary school students (US Grade 7–12 plus 1 year) in Hong Kong was insufficient [27]. In that survey, only a small portion of secondary school students knew that avulsed permanent tooth should be replanted (23.6%), or stored in cold milk (18.7%), physiological saline (24.2%) or saliva (6.7%), even they were eligible to join the school dental service and received the mentioned handbook in their primary education. Educational campaign on dental trauma management was recommended for secondary school students by the authors.

It is easy to implement poster campaign because of the low cost. In the present study, the effectiveness of dental trauma educational poster on level of knowledge was studied. Secondary school students were chosen and the cluster design was adopted since it is appropriate for the actual school environment as students might discuss with and hence influence each other. Also, since students in the same school may have some unique characteristics, e.g. higher level of health consciousness, the study was randomised at the school level to prevent contamination and improve on comparability.

## Methods

### Ethical approval

The research project was approved by the Institutional Review Board of the University of Hong Kong and Hospital Authority Hong Kong West Cluster. (HKCTR-1343, ClinicalTrials.gov: NCT01809457)

### Subjects

The subjects were secondary school students (US Grade 7–12 plus 1 year) in Hong Kong, who can read Chinese or English. We recruited the secondary schools as clusters. The protocol for this trial and supporting CONSORT checklist are available as supporting information; see Checklist S1 and Protocol S1.

### Questionnaire

The questionnaire from a survey of the same series about knowledge of dental trauma among the same target group was used [27]. Chinese and English versions of the questionnaire were constructed. There were 14 questions, divided into two sections. The first section asked for basic demographic information, whether the respondents had received formal first-aid training or acquired dental trauma information and whether they considered themselves able to distinguish between permanent and deciduous teeth. The second part consisted of questions concerning knowledge of dental traumatic injuries, which was for the assessment of dental knowledge in this study. The questionnaire was pilot tested with 59 students. Face validity, length and comprehensibility by secondary school students were pre-tested before the questionnaire was finally adopted. Face validity was established by expert opinion, and a test-retest reliability test indicated that the scores of the first and second questionnaires were positively correlated.

The marking scheme is as follows: for Q9 to Q13, 1 mark would be given for a correct answer, 0 would be given for “do not know” and 1 would be deducted for an incorrect answer. If multiple answers were chosen, 1 would be deducted for that question if an incorrect answer was chosen. There were three correct answers for Q14 as the media for storing avulsed teeth. As avulsion is the most serious type of dental trauma, timely emergency management is critical. Knowledge of more storing media raises the chance of a student being able to find one soon enough to keep the vitality of the periodontal cells on the root surface, which improves the prognosis. Therefore, 1 mark would be given for each correct answer but 1 mark would be deducted for each incorrect answer. Multiple answers were allowed.

### Poster

The educational poster was in A3 size, colourful and with pictures. One side of the poster was written in Chinese and the other side in English, The content was constructed by the authors using two publications as reference [3], [37]. This poster is the same as the one used in another study for primary and secondary school teachers of the same series [41]. Chinese and English educational posters are available as supporting information S1 and S2.

### Sample size calculation

In order to demonstrate a difference in score change of 2 marks (variance 10) between the intervention group and the control group, with a power of 90% and a statistical significance of 5%, 53 individuals are needed in each group under simple random sampling. To account for the cluster design, we assume an intracluster correlation (ICC) of 0.1. No published data on ICC under this setting could be found. However, in general practice studies, ICC takes value commonly between 0.01 and 0.05 [42], so 0.1 would be a conservative estimate. With an average of 40 students per school and a coefficient of variation of cluster size of 0.2 (after realizing that it was difficult in practice to recruit a minimum cluster size of 40 as laid down in the original protocol, we allowed clusters with size smaller than 40, but restricted the coefficient of variation of cluster size to be 0.2), the adjusted sample size is 7 schools, or 266 students, per group. To allow for potential dropouts, we aimed to recruit extra 20% individuals per group (this extra 20% was changed from 30% in the original protocol), yielding a total of 8 schools, or 319 students, per group.

### Recruitment

A staff of the principal investigator was invited to act as a voluntary secretary for this study. She was responsible for all mailings, information storage and co-ordination. She was informed that the identities of the participating schools and students should be blinded to all investigators, statistician and clerical staff at the time of appointment. She was the only one who knew the identities of the schools. She kept the information concealed and put them in a locked drawer in her room.

The Education Bureau provided a list of secondary schools upon request. There were a total of 663 schools. Special schools for intellectually disabled students were included in the list. The secretary sent invitation letters with school consent forms and individual guardian consent forms to lots of 50 randomly selected schools beginning on April 29, 2011. In each lot of invitation letters, there were 17 letters for Form 1–3 (US Grade 7–9), 17 letters for Form 4–5 (US Grade 10–11), and 16 letters of Form 6–7 (US Grade 12 plus 1 year). The contact information of the principal investigator was given in the invitation letter.

The secretary followed up with telephone calls. 16 schools with a total of 784 students joined the study after 200 invitations were sent. They replied with both signed school and individual guardian consent forms. The name and contact number of the teacher in charge were given in the school consent form.

### Randomisation and masking

The randomisation was performed after both school and individual guardian consent forms were returned. The schools were randomised to the intervention group and the control group at the school level manually using sealed envelopes. The secretary put two pieces of paper bearing the words “intervention group” and “control group” separately into two envelopes. She labelled the sealed enveloped of intervention group as group A and the other as group B. She verified that the envelope was opaque that the words could not be seen through. An independent person who did not know the details of this study were invited to assist the randomisation. The secretary labelled 1 to 16 on separate sheets of paper representing the 16 schools according to the order the consent forms were received. She folded each piece of paper and put it into an envelope and checked that the number could not be seen through, and then put them inside a box.

The independent person, not knowing the identities of group A and B, drew one envelope for group A and then one for group B alternatively until all the envelopes were drawn. The secretary then opened the envelopes and recorded the result of the randomisation. The list was put in a locked drawer that only the secretary could access.

### Implementation of the trial

The schools, teachers in charge and students were not informed of the identity of the group (intervention/control) they belonged to, educational material that they would receive and the duration of the trial. The letter of invitation and both consent forms only mentioned that the students needed to fill out two questionnaires (see protocol S1).

The trial began on May 5, 2011 and was completed on Nov 16, 2011. The first set of questionnaires was sent to both groups and hard copies were distributed to the participating students by the teachers in charge. All participating students were asked to fill out the questionnaires and returned them to the corresponding teachers in charge (in class), who then sent the completed questionnaires back to the investigator in 1 week.

A large sealed envelope containing the educational poster along with instructions was mailed to each intervention school. The teacher in charge of each school displayed the educational posters on the notice broad or at an area of similar function in the classroom. No poster was given to the control group.

The posters were removed by the teachers in charge after 2 weeks. The second set of questionnaires was then distributed to schools of both groups and the students were asked to complete the questionnaires in class. The teachers in charge then returned them to the study secretary in 1 week using prepaid envelopes. Educational posters were mailed to the control group after the completion of the study. Every procedure followed that laid down in the protocol after the trial commenced.

### Withdrawal from the study

The participating schools or individual students could withdraw from the study at any time, as mentioned in both consent forms. 39 students from the intervention group and 22 students from the control group withdrew from the study by not returning either the first or the second questionnaire.

### Data processing

The data entry staff and the statistician were blinded to the group randomisation. The statistician was instructed to analyse under the labels “group A” and “group B” according to the designed method in the protocol. The investigators were blinded to the randomisation. Only after the completion of the whole statistical report and the draft of the article, the study secretary informed the principal investigator the identities of the groups. The principal investigator then relabeled group A as intervention group and group B as control group.

### Data analysis

Individual level analysis was performed as our objective and outcome measures pertain to individual level. Our objective is to investigate the effects of the intervention, potentially controlled for some baseline information, on the gain in knowledge. The dependent variable is the score difference between the two questionnaires. To account for potential correlation among students from the same school, a linear mixed model was fitted with a normally distributed random intercept for the school effect.

To select the most appropriate model, a backward elimination method was adopted [43]. It started with including all covariates in the model: group (intervention/control), the score of the first questionnaire, gender, age, form, first-aid training, dental education in first aid, confidence in distinguishing deciduous and permanent teeth, and acquisition of dental injury information from other sources. The covariate associated with the highest p-value was eliminated in each iteration until all p-values were smaller than a threshold value 0.1.

Due to the nature of the data collected and the collection process, we anticipated that the proportion of missing data would not be high. Therefore, we simply discarded subjects who did not provide the demographic or personal information asked in the first section of the questionnaire. Missing answers for questions in the second section of the questionnaire were treated as “do not know”, and the total scores were accordingly calculated based on the marking scheme given.

The thresholds of all the statistical tests were set at 5% level of significance. The statistical analyses were performed using a computer software (JMP version 9.0.0, SAS Institute Inc., USA).

## Results

**Figure 1:**
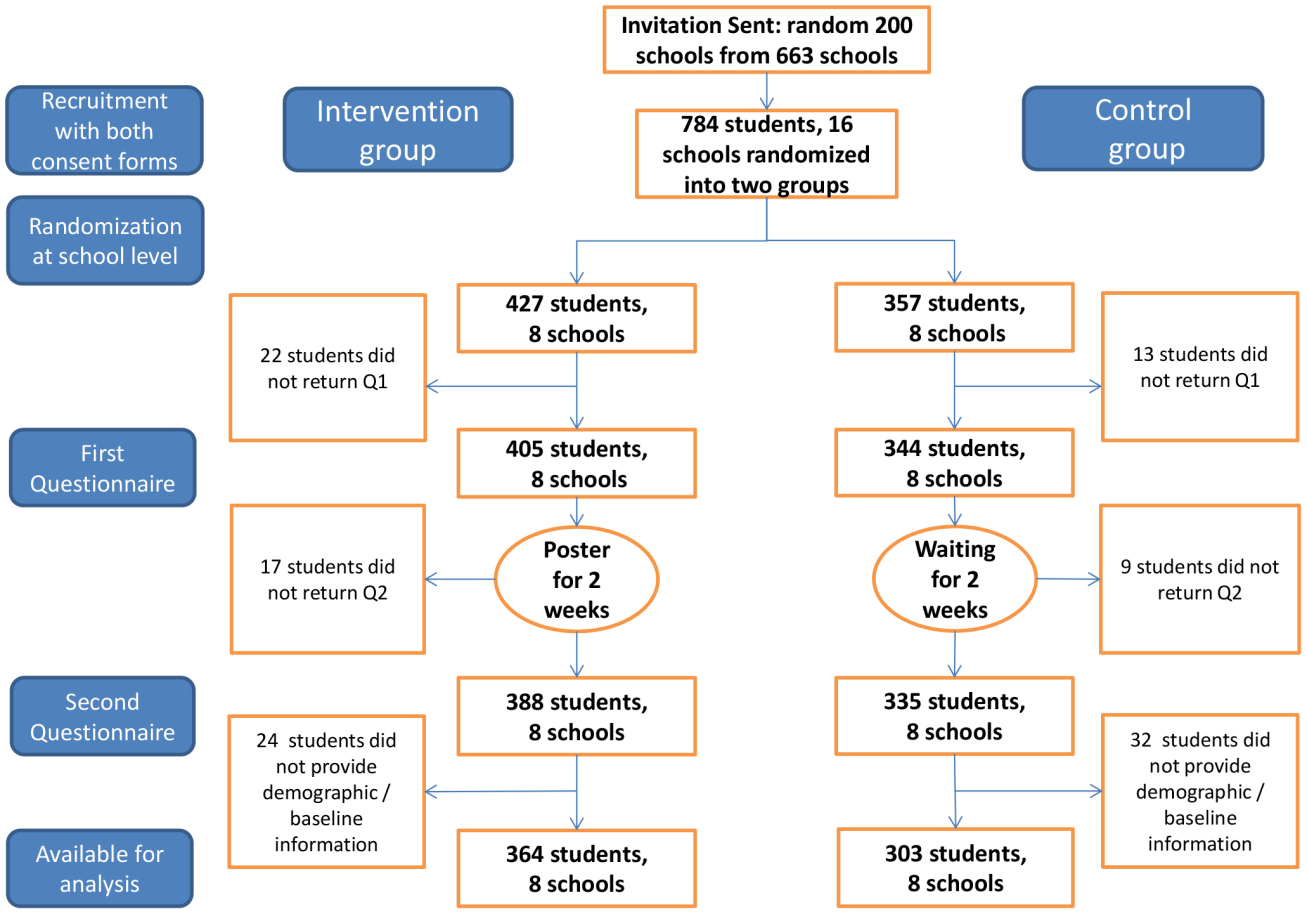
Flowchart of the participants.

There was no unintended effect or harm reported through the teachers in charge or directly to the principal investigator. After removing participants with missing background information, there were 364 individuals (8 schools) in the intervention group and 303 individuals (8 schools) in the control group available for analysis (Figure 1). The basic information for both groups on the school level and the individual level are given in Table 1. Statistical test was not conducted to compare the baseline information of the two groups [44].

**Table 1:** Demographic information and characteristics of the both groups on cluster and individual levels.

**School Level**		
	Intervention group (*n* = 8)	Control group (*n* = 8)
**Cluster Size**	Mean = 45.5; Median = 45;	Mean = 37.9; Median = 38.5;
	Min = 8; Max = 74	Min = 26; Max = 48
**Individual Level**		
	Intervention group (*n* = 364)	Control group (*n* = 303)
	Number (Percentage)	Number (Percentage)
**Gender**		
Male	122 (33.5)	113 (37.3)
Female	242 (66.5)	190 (62.7)
**Age**		
10 or below	0 (0.0)	0 (0.0)
11–13	79 (21.7)	45 (14.9)
14–16	145 (39.8)	175 (57.8)
17–19	132 (36.3)	81 (26.7)
20 or above	8 (2.2)	2 (0.7)
**Form**		
Form 1–3	220 (60.4)	111 (36.6)
Form 4–5	2 (0.5)	128 (42.2)
Form 6–7	142 (39.0)	64 (21.1)
**Received First-Aid Training**		
Yes	28 (7.7)	49 (16.2)
No	336 (92.3)	254 (83.8)
**Learnt Dental Injury Management in First-aid Training**		
Yes	4 (1.1)	10 (3.3)
No	360 (98.9)	293 (96.7)
**Confident in Distinguishing Type of Teeth**		
Yes	83 (22.8)	90 (29.7)
No	281 (77.2)	213 (70.3)
**Read or heard dental injury information besides from First-aid Training**		
Yes	101 (27.7)	91 (30.0)
No	263 (72.3)	212 (70.0)

No statistical test for comparison of baseline for both groups [43].

The average scores of each question of both questionnaires, along with the average difference in score of the two questionnaires for each group, are given in Table 2.

**Table 2:** Scores of both questionnaires of both groups.

	Intervention group (*n* = 364)		Control group (*n* = 303)	
	Number (Percentage)		Number (Percentage)	
	Baseline Score	Q2 Score	Baseline Score	Q2 Score
**Q9** Place for treatment				
Correct	126 (34.6)	116 (31.9)	118 (38.9)	102 (33.7)
Incorrect	180 (49.5)	189 (51.9)	150 (49.5)	154 (50.8)
Do not know	58 (15.9)	59 (16.2)	35 (11.6)	47 (15.5)
**Q10** Time for treatment				
Correct	216 (59.3)	207 (56.9)	168 (55.4)	150 (49.5)
Incorrect	97 (26.6)	105 (28.8)	110 (36.3)	98 (32.3)
Do not know	51 (14.0)	52 (14.3)	25 (8.3)	55 (18.2)
**Q11** Management of fractured teeth				
Correct	99 (27.2)	146 (40.1)	90 (29.7)	92 (30.4)
Incorrect	177 (48.6)	131 (36.0)	137 (45.2)	119 (39.3)
Do not know	88 (24.2)	87 (23.9)	76 (25.1)	92 (30.4)
**Q12** Management of displaced teeth				
Correct	79 (21.7)	129 (35.4)	71 (23.4)	84 (27.7)
Incorrect	180 (49.5)	139 (38.2)	175 (57.8)	129 (42.6)
Do not know	105 (28.8)	96 (26.4)	57 (18.8)	90 (29.7)
**Q13i** Management of avulsed baby teeth				
Correct	221 (60.7)	212 (58.2)	200 (66.0)	172 (56.8)
Incorrect	10 (2.7)	24 (6.6)	10 (3.3)	12 (4.0)
Do not know	133 (36.5)	128 (35.2)	93 (30.7)	119 (39.3)
**Q13ii** Management of avulsed permanent teeth				
Correct	81 (22.3)	126 (34.6)	72 (23.8)	63 (20.8)
Incorrect	126 (34.6)	95 (26.1)	110 (36.3)	95 (31.4)
Do not know	157 (43.1)	143 (39.3)	121 (39.9)	145 (47.9)
**Q14** Mediums for storage of avulsed teeth				
Mean	−0.352	0.310	−0.330	−0.261
Std. Dev.	1.117	1.466	1.155	1.077
**Total Score**				
Mean	−0.209	1.005	−0.238	−0.076
Std. Dev.	2.414	3.565	2.611	2.616
	Change = 1.214 (SD = 3.447)		Change = 0.162 (SD = 2.265)	

For Question 9–13, 1 mark for each correct answer, 0 for don't know, −1 if it is wrong or any wrong answer if chose more than 1. (−6 to 6); For question 14, 1 for each correct answer, 0 for don't know, −1 for each wrong answer (−7 to 3); Range of total score of the whole questionnaire: −13 to 9.

The result of the multiple linear regression is presented in Table 3. The covariates included in the final model were group (intervention/control) and baseline score only.

**Table 3:** Relationship between score change and intervention, baseline score.

	Estimate	Standard Error	95% Confidence Interval		p-value
**Intercept**	1.34	0.38	0.59	2.09	0.0049
**Group** * **(Intervention = 0, Control = 1)**	−1.25	0.54	−2.30	−0.19	0.0407
**Baseline Score** *	−0.40	0.04	−0.48	−0.32	<0.0001

Estimated ICC = 0.1193.

* the independent variable is significantly different from zero at 5% significance.

From the regression analysis, the group effect is significant. Given the same baseline score, individuals in intervention group had on average a score difference 1.25 (p-value = 0.0407) higher than that of individuals in control group. The baseline score effect is also significant, which indicates that an increase in baseline score of 1 mark would on average reduce the score difference by 0.40 (p-value<0.0001).

## Discussion

Effectiveness of educational poster on management of dental trauma on secondary school students was studied. From the statistical analysis, the group effect is significant. It means that the two-week display of the poster improved the score with statistical significance. Given the same baseline score, individuals in intervention group had on average a score difference 1.25 (p-value = 0.0407) higher than that of individuals in control group. However, a score difference of 1.25 marks is smaller than our expectation.

Some questions were not answered any better in the intervention group after the two-week display of poster. It may be that students understood and/or remembered selective portion of the information. Students may be more interested in reading certain area of the poster and may not have gone through the entire poster.

This result reflects whether the students had read, understood and remembered information on the poster. This is the first cluster randomised controlled trial for investigating the effectiveness of educational posters on dental trauma on this age group.

Since a model containing all relevant information collected in the questionnaire is too large under the current sample size, which would make the estimation unstable, we have chosen to adopt a backward variable selection procedure. We are aware that such method would possibly inflate type I error due to multiple testing, and the significant factors remained in the model may just be chosen by coincidence. It means that the p-values of the factors in the model tend to be smaller and the confidence intervals tend to be narrower than they, strictly speaking, should be. One should bear that in mind when interpreting the results. However, under the current setting, the major factor of interest is the intervention effect, which would not be eliminated under the variable selection procedure. The problem of concluding a significant intervention effect by coincidence as a result of variable selection does not exist. Also, besides intervention, there is only one factor remaining in the model, namely the baseline score. Therefore, the inflation of Type I error, if any, is minimal.

With the above being said, one may still be interested in the estimated marginal effect of intervention. Fitting a linear mixed model with only intervention group as independent variable, the estimated effect is 1.26 (p-value = 0.0423), which means that on average students from the intervention group had score improvement of 1.26 more than that of students from the control group. It is, though marginally, significant at 5% level of significance.

The immediate effect of two-week display of poster was investigated, while long term effect is out of the scope of this study. Knowledge, rather than the management of traumatic dental injuries, was tested because long term follow up is necessary for the latter. It is not feasible to carry it out in Hong Kong because the number of cases from the sampling frame may not be large enough to produce a sizable sample that would produce statistically significant results. The list of all primary and secondary schools was exhausted even for the series of short term studies.

From the 663 secondary schools in Hong Kong, with altogether 454244 students, the sample was randomly selected with only the condition that the students were able to read Chinese or English. The results apply to all these students. The generalizability of the results to other countries is unclear since the culture, students' workload, educational system, health consciousness, ability to comprehend the study information and the importance students placed on dental trauma material may differ.

As some schools display a lot of information to students and change the notices or posters quite frequently, display time of longer than two weeks may not be feasible. Classroom is the most suitable location for effectively displaying information to students.

Though they are usable media for storage, Hank's balanced salt solution (or e.g. Save-A-Tooth), Viaspan, eagle's medium and propolis culture medium were not mentioned in the choices explicitly in question 14 because these were not accessible to students in Hong Kong. However, if students mentioned these in the “others (please specify)” option, they would be considered correct. Nevertheless, no student mentioned any of these solutions. These media were not mentioned in the poster for the same reason.

The randomisation of this trial was blinded to the investigator, data entry staff and the statistician. Only the secretary knew the identities of group A and B, and this information was given to the investigators only after the whole statistical report and the manuscript were drafted. Other than relabeling “group A” and “group B” as “intervention group” and “control group”, respectively, no information on the figure or results was amended. So doing was to minimize bias and to improve the representability of the statistical analysis result.

Educational posters are relatively inexpensive and easy to distribute. There is no temporal limitation and assembly of students is not needed, as in the case of lectures and seminars. Displaying educational posters in classrooms is practical and effective means to improve students' knowledge of dental trauma.

## Conclusion

Educational poster statistically significantly improves the student's knowledge of emergency management of dental trauma.

## Supporting Information

Checklist S1 (DOCX)

Poster S1 **Chinese Educational poster.**
(PDF)

Poster S2 **English Educational poster.**
(PDF)

Protocol S1 (PDF)
