# Supplementary material for: Effectiveness of Educational Poster on Knowledge of Emergency Management of Dental Trauma - Part 2: Cluster Randomised Controlled Trial for Secondary School Students
Source: PLoS One. 2014 Aug 5;9(8):e101972. doi: 10.1371/journal.pone.0101972 (PMC4122350; doi:10.1371/journal.pone.0101972)
Supplement: Protocol S1 — (PDF) [file pone.0101972.s004.pdf]

香港大學
University of Hong Kong

醫院管理局
HOSPITAL AUTHORITY

香港大學及醫管局港島西醫院聯網研究倫理委員會
Institutional Review Board of the University of Hong Kong/
Hospital Authority Hong Kong West Cluster (HKU/HA HKW IRB)
Address: Rm 901, Administration Block, QMH Tel 2255 3923 2255 4086 Fax 2255 4735

Dr. Cecilia Young
105A, 1/F Liberte Place, 833 Lai Chi Kok Rd, KLN

18-Apr-11

Dear Dr. Young,

IRB Reference Number: **UW 11-177**

The HKU/HA HKW IRB is authorized by a joint agreement of the University of Hong Kong and Hospital Authority Hong Kong West Cluster to review and monitor clinical research. It serves to ensure that research complies with the Declaration of Helsinki and acts in accordance to ICH GCP guidelines, local regulations and Hospital Authority and the University policies.

I write to inform that your research application/submission has been approved by an expedited process with details shown below. You are also requested to adhere to the conditions listed.

**IRB reviewer(s)**	:	Professor CL Lai, Deputy Chairman and Professor KM Chu, Deputy Chairman of the HKU/HA HKW IRB
**Protocol title**	:	Effectiveness of educational poster to secondary students regarding emergency management of dental trauma - A cluster randomised controlled trial
**Study site(s)**	:	As stated in application form
**Document(s) approved**	:	01. Clinical research ethics review application form
	:	02. Protocol (S01 26.3.2011)
	:	03. Information sheet (25.3.2011) - English and Chinese
	:	04. School's Informed Consent Form (25.3.2011) - English and Chinese
	:	05. Guardian's Informed Consent Form (25.3.2011) - English and Chinese
	:	06. Questionnaire - English and Chinese
	:	07. Educational Posters - English and Chinese
**Document(s) reviewed**	:	08. Short CV of principal investigator

(Conditions : 1. Do not deviate from, or make changes to the study protocol without prior written IRB approval, except when it is necessary to eliminate immediate hazards to research subjects or when the change involves only logistical or administrative issues.

2. Report the following to HKU/HA HKW IRB: (i) study protocol or consent document change (use 'HKU/HA HKW IRB RE001F7'), (ii) serious adverse event (use 'HKU/HA HKW IRB RE001F8'). (iii) study progress (use 'HKU/HA HKW IRB RE001F9a')* (iv) new information that may be relevant to a subject's willingness to continue participation in the study.

3. Report study progress to HKU/HA HKW IRB at a 12-monthly interval until study closure.)

Yours sincerely.

W. H. Lee
Secretary, HKU/HA HKW IRB

Protocol

S01 26.3.2011

Effectiveness of Educational Poster to Secondary Students regarding Emergency Management of Dental Trauma - A cluster randomised controlled trial

Background

Dental Injuries is common, if the patient or person next to him/her acts correctly and fast, the damage will be minimized and most teeth and tissue could be saved.

Secondary students are physically active. School is a good place where students can read posters in classrooms easily, if they know how to deal with dental injuries, it is good for themselves and people they meet.

A poster placed in the notice board in classroom can be easily seen and read by students. It can be placed for a long time, it might be a good method to deliver the knowledge.

Aim

To investigate the effectiveness of the educational poster about dental trauma to increase the knowledge of secondary students.

Methodology

Schools will be randomly divided into two groups, baseline score of knowledge in questionnaire will be collected for all subjects. Poster will be sent to intervention group, the school will place it at the notice broad or area of similar function in the classroom. For the control group, nothing will be given, then after 2 weeks, the score of knowledge by the second questionnaire of both groups will be collected again.

To compare the difference in knowledge before and after in both groups, statistical test will be performed to show it is not due to chance.

Design

We conduct a cluster randomized controlled trial. A list of secondary schools was requested from the Education Bureau. Secondary schools will be invited to join the study and then randomized to the intervention group and the control group in the unit of school using computer generated numbers. All students will fill in the first questionnaire and send it back to the investigator in 1 week. Then copies of the same poster will be given to the intervention schools, these schools will be requested to

place them in the classrooms of the participating students. No poster will be given to the control group. After 2 weeks, the poster will be removed. The same questionnaire will be filled out by all the students in both groups and sent back in 1 week. Then the data will be processed to show whether the poster is effective to improve the knowledge of the students.

This study will comply with Declaration of Helsinki and ICH GCP guidelines.

Eligibility

All secondary schools in Hong Kong that the students can read Chinese or English.

Withdrawal of students from the study

The participating students can withdraw from the study at any time.

Sample size determination:

In determining the k, the number of cluster required to achieve a power of (1-β) and a significance of α, we consider the following formula [1]:

$$k = \frac{(z_{\alpha/2} + z_{\beta})^2 (2\sigma^2)[1 + (m-1)\rho]}{m(\mu_1 - \mu_2)^2}$$

In this formula, $z_{\alpha/2}$ and z_{β} are standard normal distribution values corresponding to upper tail probabilities of α/2 and β respectively. m is the cluster size, ρ is the intracluster correlation coefficient. $|\mu_1 - \mu_2|$ is the minimum difference in outcomes of intervention and control groups which can be regarded as significant, and σ is the standard deviation of the outcome variable.

In order to demonstrate a difference in score improvement of 2 marks (variance 10) between the intervention group and the control group, with a power of 90% and a statistical significance of 5%, 53 individuals are needed in each group under simple random sampling. To account for the cluster design, we assume an intracluster correlation (ICC) of 0.1. No published data on ICC under this setting can be found. However, in general practice studies, ICC take values commonly between 0.01 and 0.05 [2], so 0.1 would be a conservative estimate. With an average of 40 students recruited per school, the adjusted sample size is 7 schools, or 258 students, per group. To allow for potential dropouts, we aimed to recruit extra 30% individuals per group, yielding a total of 9 schools, or 335 students, per group.

To obtain a better estimate for the variance, we will conduct a pilot test with two schools (with about 80 students). In the pilot test, we ask each student to fill out the questionnaire once. We estimate the variance of the difference in score improvement between the intervention group and the control group by two times the sample variance of the scores obtained from the pilot test. The sample size will then be adjusted according to the new estimated variance according to the pilot test.

Recruitment

We send invitation letters with school consent form and guardian consent form to lots of 50 schools randomly from the schools in the above-mentioned school lists until the desired sample size is achieved. We aimed at 18 schools, with each school having at least 40 students participating. We will then randomize them to the intervention group and control group in the unit of school. The sample size will be finalized after the pilot test from the 2 schools that are first to reply, one will be for control and one for intervention.

Statistical Analysis

Only students who have completed both questionnaires before and after the study will be included in the analysis. To accommodate possible dependence between observations from the same school, multilevel regression will be conducted to investigate the changes of questionnaire score before and after the study. In the regression model, the score change will be considered as the dependent variable. The group (intervention/control), baseline questionnaire score, age, gender, and interaction terms between group and baseline questionnaire score, age and gender are considered as potential factors that will be included in the analysis. Significance of the regression coefficients will be tested at 5% level of significance. All tests are two-sided.

Reference

1. *Impact of educational posters on the lay knowledge of school teachers regarding emergency management of dental injuries Dental Traumatology 2009*; 25: 406–412;
2. Allan Donner and Neil Klar. *Design and Analysis of Cluster Randomization Trials in Health Research.* London: Arnold, 2000.
3. Underwood M, Barnett A, Hajioff S. *Cluster randomization: a trap for the unwary.* Br J Gen Pract 1998; 48: 1089-1090.

21.4.2011

Dear PTA Chairperson, teachers and guardians,

Ref: Academic Study for dental injuries – questionnaire for students

I am a public health practitioner and researcher, I am now co-operating with Prof Cheung Lim Kwong and Mr Alex Wong in an academic study for dental injuries. This is a joint project with the University of Hong Kong. We invite your school to join this study.

Students of the participating schools will be the first group to learn the essential but simple and precise steps of how to deal with dental injuries in accidents before professional care are provided. This will benefit your students and save more teeth.

The result of this study will give the dental field some information about whether the educational poster for secondary students about dental injury improves student's knowledge. It can be comprehended in around **10 minutes**. Also, all secondary schools in Hong Kong will ultimately receive the same educational poster if the result is positive.

We cannot proceed to the printing and sending of the posters without this study. Your participation is important to all secondary schools. We will acknowledge all participating schools in a list when we send the posters. Please tell us if you do not want your school appears in the list.

Participating students will receive altogether **2 sets of questionnaire** which consist of 14 MC questions which can be filled in around **7 minutes**. They are basic data and dental injury knowledge questions. There will be no sensitive questions and all information will comply with rules in Hong Kong and international medical research ethics code.

We gave the guardian's consent forms, questionnaires and posters to some F.X students and their guardians; they filled out, returned and told us that this topic was highly related to their daily life. They hope this study could be completed and posters could be sent to all secondary schools in Hong Kong.

Please sign the **school consent form** and **individual guardian's consent form** if you can help. Completion of 2 sets of questionnaires is necessary to give the data for the analysis. **However, students have the absolute right to withdraw at any time even they signed the consent form.** We need at least **40 students (F.X-F.Y)** in each participating school.

We need your support. Please discuss with your collegues and guardian's representatives, Please mail the consent forms with the prepaid envelope best before **7.5.2011.** Kindly contact Cecilia Young at 23618684 if you have any enquiries or if you want more time.

Thank you for your kind attention.

Cecilia Young (all documents were sent to the Principal)

Research Team – Poster effectiveness – Secondary Student Trial

105A, 1/F Liberte Place, 833 Lai Chi Kok Road, Kln Tel 23618684 ceciliatyp@yahoo.com.hk

21.4.2011

親愛的校長、老師及家長：

醫學研究 — 牙齒創傷

香港大學的張念光教授及黃堅祐先生與我現進行牙齒創傷的醫學研究，我們邀請貴校參與。

參與學生是全港第一批接觸有關牙齒創傷即時處理方法的中學生，內容包括傷者於正式醫治前簡單而必需的處理步驟，正確方法能救回更多牙齒。

是次研究目的為測試教育性海報能否提高中學生牙齒創傷方面的知識，只需大約十分鐘閱讀。本研究將給予牙醫學界一些數據，如牙齒創傷資料能有效傳達知識，全港所有中學都會收到相同教育性海報。

印制及寄出海報需以此研究結果為基礎，如此研究不能完成，我們無法寄出海報，貴校的參與對所有中學十分重要。我們在寄出海報時會鳴謝參與的學校，如不希望列出貴校，請告知我們。

參與學生需填**二份選擇題問卷**，有十四條選擇題，大約需要七分鐘，內容為基本資料及牙齒創傷問題，不涉及敏感問題，並會依從所有香港港法例及國際性的醫學研究倫理守則（包括保密及其他）。

我們曾把學生家長同意書、問卷及海報給予一些中一學生及監護人，他們均認為牙齒創傷資料對日常生活有用，希望研究完成，全港中學都收到此海報

每間參與學校需要**最少 40 名學生**。請校長或負責老師隨意選中 X 至中 X 任何一至數班同學 (如一班不足，便邀請兩班，多於 40 人即可)，如中一至中二沒有足夠同學，次選其他級別 (混合級別亦可)。我們希望不同學校盡量有不同級別。

請參與本研究，填寫一份**校方同意書**及所有參與同學的**學生家長同意書**。參與學生需填二份選擇題問卷，研究人員需要二份結果用作分析。**但參與學生即使已簽署同意書仍有絕對權利退出研究。**

我們需要您的支持! 請參與是次研究。敬希盡早以 **freepost** 回郵信封寄回**兩種同意書**。**7.5.2011** 前最佳，如需更多時間或有查詢請電 23618684 或 電郵 ceciliatyp@yahoo.com.hk 楊小姐，謝謝！

研究團隊　　　　　　(所有文件已經寄給校長)

School's Informed Consent Form

Effectiveness of educational poster to secondary students regarding emergency management of dental trauma - A cluster randomised controlled trial

1. The aim of this study is to test whether the educational poster can improve the secondary students' knowledge in dental injuries.

2. There will be 2 sets of questionnaires. Participating students need to fill out both of them. We need to analyze both questionnaires.

3. There are 14 multiple questions in the questionnaires. They consist of basic data and dental injury knowledge. There will be no sensitive questions and all information will comply with all rules in Hong Kong (keep confidential and other law) and international medical research ethics code.

4. Participating students are the first group of secondary students who receive guidelines of handling of dental injuries in Hong Kong.

5. The data are used for academic study for whether educational poster to secondary students improves students' knowledge. It gives the dental field some information. All secondary schools in Hong Kong will receive the same educational poster if the result is positive. The printing and sending can only be proceeded with the basis of this study.

6. **Participating student has the absolute right to withdraw this study at any stage.**

7. A copy of this consent form will be given to the school.

Participating school ______________________________________

Tel:

Number of classes:

Date:

Contact person (print name):

____

Authorized signature with school chop

校方知情同意書

牙齒創傷教育性海報提高中學生知識的效能-群組隨機對照試驗

1. 研究目的為測試教育性海報能否提高中學生牙齒創傷方面的知識。

2. 參與學生需填二份選擇題問卷，研究人員需要二份結果用作分析。

3. 問卷有十四條選擇題，內容為基本資料及牙齒創傷問題，不涉及敏感問題，並會依從所有香港法例及國際性的醫學研究倫理守則（包括保密及其他）。

4. 參與學生是全港第一批接觸有關牙齒創傷處理方法的中學生。

5. 結果用作學術研究用途，以調查所提供的資料能否提高學生的知識。本研究將給予牙醫學界一些數據，如牙齒創傷海報能有效傳達知識，全港所有中學均會收到相同教育性海報。印刷及郵遞海報必需在研究完成後進行並以此為基礎。

6. **參與學生即使已簽署同意書仍有絕對權利退出研究。**

7. 本同意書副本將給予參與校方。

參與學校：________________________________

電話　：____________

數目：_____ 班

日期　：____________

負責老師：____________

____

校方蓋印

Guardian's Informed Consent Form

Effectiveness of educational poster to secondary students regarding emergency management of dental trauma - A cluster randomised controlled trial

1. The aim of this study is to test whether the educational poster can improve the secondary students' knowledge in dental injuries.

2. There will be 2 sets of questionnaires. Participating students need to fill out both of them. We need to analyze both questionnaires.

3. There are 14 multiple questions in the questionnaires. They consist of basic data and dental injury knowledge. There will be no sensitive questions and all information will comply with all rules in Hong Kong (keep confidential and other law) and international medical research ethics code.

4. Participating students are the first group of secondary students who receive guidelines of handling of dental injuries in Hong Kong.

5. The data are used for academic study for whether educational poster to secondary students improves students' knowledge. It gives the dental field some information. All secondary schools in Hong Kong will receive the same educational poster if the result is positive. The printing and sending can only be proceeded with the basis of this study.

6. **Participating student has the absolute right to withdraw this study at any stage.**

7. A copy of this consent form will be given to each participating student's guardian.

I can read English.

Student's Print Name: **Class:**

School: ____

Please give Chinese / English questionnaire and poster to student.
(Please cross out the unsuitable language)

Guardian's Print Name:

____

Signature
Date:

學生家長知情同意書

牙齒創傷教育性海報提高中學生知識的效能-群組隨機對照試驗

1．研究目的為測試教育性海報能否提高中學生牙齒創傷方面的知識。

2．參與學生需填二份選擇題問卷，研究人員需要二份結果用作分析。

3．問卷有十四條選擇題，內容為基本資料及牙齒創傷問題，不涉及敏感問題，並會依從所有香港港法例及國際性的醫學研究倫理守則（包括保密及其他）。

4．參與學生是全港第一批接觸有關牙齒創傷處理方法的中學生。

5．結果用作學術研究用途，以調查所提供的資料能否提高學生的知識。本研究將給予牙醫學界一些數據，如牙齒創傷資料能有效傳達知識，全港所有中學均會收到相同教育性海報。

6．參與學生即使已簽署同意書仍有絕對權利退出研究。

7．本同意書副本將給予參與學生家長。

本人能閱讀中文。

參與學生姓名(正楷): **班別及學號:**

學校: ________________________________

家長或監護人姓名:

____

簽署

日期:

Name: ________________ **X Questionnaire**

School: _________________

Date:

Effectiveness of educational poster to secondary students regarding emergency management of dental trauma - A cluster randomised controlled trial

Part 1 demographics

1. Gender: ○a. Male ○b. female

2. Age: ○a. under 10 years old ○b. 11-13 years old ○c. 14-16 years old ○d. 17-19 years old ○e. 20 or above

3. Which grade are you studying?
   ○a. F.1-F.3 ○b. F.4-F.5 ○c. F.6 – F.7

4. Have you received any formal first aid training?
   ○a. Yes (go to Q5) ○b. No (go to Q6)

5. Is the first aid about dental injury included in your formal first aid training?
   ○a. Yes ○b. No

6. Do you think that you can distinguish a primary tooth (baby tooth) from a permanent tooth by considering the patient's age and looking at the tooth, the socket or the position?
   ○a. Yes ○b. No

7. Have you ever received any educational material/information about the injury except formal first aid training (before this study)?
   ○a. Yes (go to Q.8) ○b. No (go to Q.9)

8. Which way?
   ○a. TV ○b. Journals ○c. Radio ○d. School
   ○e. Poster ○f. Newspapers ○g. Internet ○h. Oral description
   ○i. Others (please specify) ___________________

Part II knowledge

9. If there is a dental injury, the impact site is the teeth, only the teeth and surrounding part seemed to be injured, what should the patient do?
- ○ a. Go to the casualty in the nearest hospital on foot or by any transport
- ○ b. Call an ambulance; go to the casualty in the nearest hospital.
- ○ c. Go to the nearest private doctor
- ○ d. Go to the patient's family doctor
- ○ e. A dentist
- ○ f. Treat it by self
- ○ g. Others (please specify)____________________
- ○ h. Don't Know

10. When should the patient go or be brought for the examination and treatment?
- ○ a. At lunch or after school
- ○ b. After plenty of rest
- ○ c. Within 24 hours
- ○ d. Within 48 hours
- ○ e. When the parent or guardian is free to bring the patient for examination and treatment
- ○ f. Any time when the patient feels relax and want to have treatment
- ○ g. Within 4 hours
- ○ h. Immediately
- ○ i. Others (please specify) ____________________
- ○ j. Don't Know

11. If the tooth is fractured, the patient or surrounding person should:
- ○ a. The fractured part is useless, ignore it
- ○ b. Try to find the fractured part, wrap it with gauze or tissue and bring it for examination and treatment
- ○ c. Put it in liquid medium and bring it for examination and treatment
- ○ d. Others (please specify) ____________________
- ○ e. Don't Know

12. If the tooth is displaced or extruded but still attached, the patient or surrounding person should:

○ a. Do not touch, let it remains its new position

○ b. Try to put back to the original position

○ c. Ask the patient to carefully clench one's teeth if it is possible

○ d. Others (please specify) ____________________

○ e.. Don't Know

13. Should a knocked out teeth be put back to the original position?

Baby tooth	○a. Yes	○b. No	○Don't know
Permanent tooth	○a. Yes	○b. No	○Don't know

14. If a tooth is knocked out? It should be: (1 or more)

○ a. The tooth is useless, do not spend time to find it or to work on it

○ b. Wrap it in gauze or tissue and bring it to examination and treatment

○ c. Soak it in cold milk in a container and bring it to examination and treatment

○ d. Soak it in physiological saline in a container (from pharmacy) and bring it to examination and treatment

○ e. Soak it in the patient's saliva in a container and then bring the container to examination and treatment

○ f. Soak it in tap water in a container and bring the container to examination and to treatment

○ g. Soak it in distilled water in a container and bring the container to examination and to treatment

○ h. Put it in a container or a plastic bag in dry condition and bring it to examination and treatment

○ i. Soak it in a container with disinfectant solution and bring it to examination and treatment

○ j. Others (please specify) ____________________

○ k.. Don't Know

End of the questionnaire

同學姓名(正楷) ______________ 班別及學號 _______
學校: ________________________________
日期

牙齒創傷教育性海報提高中學生知識的效能-群組隨機對照試驗

第一部份 基本統計資料

請在適當橫線上填上✓號。

1. 性別 ： ○ a. 男 ○ b. 女

2. 年齡 ： ○ a. 十歲或以下 ○ b. 十一至十三歲 ○ c. 十四至十六歲
○ d. 十七至十九歲 ○ e. 二十歲或以上

3. 班級 ： ○ a. 中一至中三 ○ b. 中四至中五 ○ c. 中六至中七

4. 你曾否接受過正規急救訓練？
○ a. 有，請列明 ___________（往第 5 條） ○ b. 沒有（往第 6 條）

5. 正規急救課程內容有否包括牙齒創傷？
○ a. 有 ○ b. 沒有

6. 你認為自己是否能從傷者年齡，觀察受傷牙齒外形、牙床及其位置分辦出乳齒或恆齒？
○ a. 是 ○ b. 不是

7. 除正式急救外，在這次調查以前，你曾否閱讀或收聽過任何有關牙齒創傷的教育資料？
○ a. 有 （往第 8 條） ○ b. 沒有（往第 9 條）

8. 你從哪種途徑得悉有關牙齒創傷的教育資料？
○ a.電視 ○ b.雜誌 ○ c.電台 ○ d.學校
○ e.海報 ○ f.報紙 ○ g.互聯網 ○ h.口頭描述
○ i.其他（請列明）__________________

第二部份 有關知識

9. 如果有意外，以致牙齒創傷，其受傷位置只在牙齒及附近口腔位置，傷者應怎樣做？

○ a. 步行或以交通工具往最近的醫院急症室
○ b. 召救傷車，往最近醫院急症室
○ c. 往最近的私人執業全科醫生
○ d. 往傷者的家庭醫生
○ e. 往牙醫
○ f. 自行處理
○ g. 其他（請列明）____________________
○ h. 不知道

10. 傷者應何時前往或被送往診治？

○ a. 午膳或放學後
○ b. 充分休息後
○ c. 二十四小時內
○ d. 四十八小時內
○ e. 當家長或監護人有空帶傷者前往診治時
○ f. 當傷者放鬆心情並希望診治時
○ g. 四小時內
○ h. 立刻
○ i. 其他（請列明）____________________
○ j. 不知道

11. 如牙齒斷裂，傷者或附近人士應：

○ a. 斷裂部份無用，不需理會
○ b. 找出斷裂部份，以紗布或紙巾包裹帶往診治
○ c. 把斷裂部份放入液體內，帶往診治
○ d. 其他（請列明）____________________
○ e. 不知道

12. 如牙齒依然留在牙床內但移位或拉出了一些，傷者或附近人士應：

○ a. 不應接觸，保持現在新的位置
○ b. 嘗試用手指把它移回原位
○ c. 如有可能，請傷者小心慢慢地咬合
○ d. 其他（請列明）____________________
○ e. 不知道

13. 碰撞後飛脫的牙齒是否應該放回牙床原來位置？

乳齒	○應該	○不應該	○不知道
恆齒	○應該	○不應該	○不知道

14. 如牙齒碰撞後飛脫，應：（可選擇一個或以上）

○ a. 牙齒沒有用處，不應浪費時間找尋及處理
○ b. 以紗布或紙巾包裹，帶往診治
○ c. 放入凍牛奶內，浸過牙齒，帶往診治
○ d. 放入生理鹽水內（從藥房買來的），浸過牙齒，帶往診治
○ e. 放入盛有傷者的口水的器皿內，浸過牙齒，帶往診治
○ f. 放入盛有自來水的器皿內，浸過牙齒，帶往診治
○ g. 放入盛有蒸餾水的器皿內，浸過牙齒，帶往診治
○ h. 放入器皿或膠袋內，乾的狀態下，帶往診治
○ i. 放入盛有消毒藥水的器皿內，浸過牙齒，帶往診治
○ j. 其他（請列明）____________________
○ k. 不知道

問卷完
